# Neostigmine^®^ Improves Pancreatic Duct Visualization in Magnetic Resonance Cholangiopancreatography and Could Be a Cheap Alternative for Secretin

**DOI:** 10.5152/tjg.2022.21864

**Published:** 2022-08-01

**Authors:** Paulina F. Toledo, Gonzalo Cárdenas, Zoltán Berger, Daniela Simian, Francisca Araya

**Affiliations:** 1 Division of Gastroenterology, Department of Internal Medicine, Hospital Clínico Universidad de Chile, Santiago, Chile; 2 Department of Radiology, Hospital Clínico Universidad de Chile, Santiago, Chile

**Keywords:** Magnetic resonance cholangiopancreatography, Neostigmine®, pancreatic ducts, secretin

## Abstract

**Background:**

To determine the effect of intramuscular administration of Neostigmine^®^ on the visualization of the pancreatic duct on magnetic resonance cholangiopancreatography in patients with recurrent acute pancreatitis or abdominal pain.

**Methods:**

We reviewed patients undergoing magnetic resonance cholangiopancreatography followed by a Neostigmine^®^-enhanced magnetic resonance cholangiopancreatography. Patients with a history of recurrent acute pancreatitis or abdominal pain who had a magnetic resonance cholangiopancreatography where the pancreatic duct was not entirely seen, were selected to undergo a second magnetic resonance cholangiopancreatography 40 minutes after 0.5 mg Neostigmine^®^. Images were analyzed by 2 radiologists. The diameter of the pancreatic duct was measured in the head, body, and tail of the pancreas on the baseline images and after Neostigmine^®^.

**Results:**

Ten patients were included, with a median age of 33 years (range 15-61). The maximum diameter of the pancreatic duct increased significantly after Neostigmine^®^ administration in all patients, from 1.84 ± 0.98 to 3.41 ± 1.27 mm in the head, 1.34 ± 0.42 mm to 2.5 ± 0.49 mm in the body and 0.72 ± 0.52 mm to 1.78 ± 0.43 mm in the tail (mean ± SD, *P* < .0001). Neostigmine^®^ helped to provide better detail of the pancreatic duct anatomy in 4 patients. In 2 patients we confirmed pancreas divisum, in another the Santorini duct was not seen on the baseline images but it was clearly visualized after Neostigmine^®^, and in the fourth patient, Neostigmine^®^ improved visualization of multiple pancreatic duct stenosis.

**Conclusion:**

Neostigmine^®^-magnetic resonance cholangiopancreatography significantly increases the diameter of the pancreatic duct, allowing an accurate morphological evaluation. It could be a cheap alternative to secretin, which is expensive and hardly available.

**Main Points**

- While magnetic resonance cholangiopancreatography is an excellent method, pancreatic ducts are not clearly seen in some cases.
- Stimulation of pancreatic secretion results in increased fluid content in the ducts and as a consequence, better visualization of the ducts.
- Neostigmine^®^ is a cheap alternative to the expensive and hardly available secretin and allows the detection of different anomalies in the duct system.

## Introduction

The diseases of the pancreas can be diagnostically challenging, even by current imaging modalities.^1^ Magnetic resonance cholangiopancreatography (MRCP) allows non-invasive evaluation of the biliary and pancreatic duct (PD) with heavily T2-weighted sequences, which highlight fluid-filled structures.^2^ In the past, endoscopic retrograde cholangiopancreatography (ERCP) was the mainstay in the diagnosis of PD pathology; however, it is not only expensive and time-consuming but also adds a significant risk of morbidity and even mortality.^3^ In consequence, nowadays it is reserved for therapeutic purposes.

Visualization of the PD is important for the detection and characterization of several pancreatic disorders since many pancreatic diseases involve or even originate from the PD.^4^ However, in physiological conditions, the pancreatic ducts are not always easily recognizable.^3-5^ Stimulation of pancreatic secretion increases the quantity of fluid in the duct system and improves the visualization of anatomical details of the PD. In this way, the use of intravenous secretin-enhanced in MRCP (S-MRCP) to induce fluid production from the pancreas, is widely accepted to enhance the visualization and evaluation of the PD system.^6-8^ Moreover, it improves the detection of congenital variants of the pancreaticoduodenal junction and facilitates imaging of the PD after most common pancreatic surgical procedures and it can detect subtle anomalies in the PD, increasing the sensitivity of the diagnosis of chronic pancreatitis in earlier phases, providing also semiquantitative information about the pancreatic function.^6^ However, S-MRCP is performed only in a few centers due to its high cost and limited accessibility. Its availability and cost render practically impossible its routine use in Chile and probably in many other countries. Given this, an attempt has been made to find alternatives.

Prostigmine (Neostigmine^®^ [Fresenius Kabi, USA; Lake Zurich, IL, USA]), is a reversible acetylcholinesterase inhibitor, acts by increasing the level of acetylcholine (Ach) and increases the pancreatic fluid and enzyme secretion.^9^ The present study aimed to assess the impact of the administration of Neostigmine^®^ in improving the imaging quality of the pancreatic ducts in MRCP in subjects with a history of recurrent pancreatitis or abdominal pain of uncertain etiology.

## Materials and Methods

The project was approved by the local Committee of Scientific Ethics (number 38/20, July 15, 2020).

An observational, single-center retrospective study was undertaken where MRCP-enhanced with Neostigmine^®^ was searched in our database and evaluated over a 5-year period from January 2015 to December 2019. Magnetic resonance cholangiopancreatography has been routinely performed in patients with recurrent acute pancreatitis or abdominal pain suggestive of pancreaticobiliary disease, with the exception of individuals with contraindications for magnetic resonance image (MRI) (cardiac pacemaker, brain aneurysm clips, cochlear implants, claustrophobia). Those with a baseline MRCP where the pancreatic ducts were not completely visualized, were selected to undergo a second MRCP 40 minutes after an intramuscular injection of 0.5 mg Neostigmine^®^. Patients with contraindications for Neostigmine^®^ (peritonitis, mechanical obstruction of the intestinal or urinary tract, and hypersensitivity to this class of compounds) were excluded.

The patients were observed till 3 hours after the injection and contacted by phone call 24 hours after the MRCP to detect any drug-related side effects.

## Equipment and Technique

All MRCP were performed with a 1.5 T Symphony Master Class system (Magnetom Symphony; Siemens, Erlangen, Germany). Magnetic resonance image acquisitions were made according to the institution’s acquisition imaging protocol shown in Table 1. Magnetic resonance cholangiopancreatography was performed after at least 6-hour fasting, acquiring images with 2-dimension and 3-dimension (3D) techniques. A second MRCP was taken on the same day, after 40 minutes of an intramuscular injection of 0.5 mg of Neostigmine^®^^,^ using the same protocol as the baseline exam.

## Image Analysis

The images were evaluated by 2 abdominal radiologists, with 8 and 6 years of experience in gastrointestinal radiology. The images were filed in DICOM format and were arranged on high-resolution displays utilizing the AGFA IMPAX 6.6.1 medical imaging analysis software system.

The 3D MRCP images were initially evaluated, also utilizing the millimetric sections of the source images and multiplanar reconstructions with the maximum intensity projection technique. Quantitative assessment was based on evaluation by the naked eye. The diameters of the pancreatic ducts were measured in the head, body, and tail at the point in each segment with the maximum diameter on axial T2-weighted sequences. The maximum diameter of the PD segments was measured in millimeters before and after Neostigmine^®^ administration by the 2 experienced radiologists. When the pancreatic duct was not visualized, the diameter of the duct was considered as 0 mm. A stricture was considered when the pancreatic duct abruptly changed its caliber by more than 1 mm.

The 2 expert radiologists made the measurements independently of both conventional MRCP and N-MRCP. They registered any visible pancreatic duct anomaly with conventional MRCP and after the use of Neostigmine^®^.

## Statistical Analysis

Grouped data are represented as median and range or mean values ± standard deviation according to their distribution. For statistical comparisons, we used paired t-test to analyze the difference in the diameter of each segment of the pancreatic duct before and after Neostigmine^®^ administration. Intraclass correlation coefficient (ICC) was calculated between the 2 radiologists’ measurements, considering correlation <0.40 poor, 0.40-0.60 fair, 0.60-0.75 good, and >0.75 excellent. A *P*-value of less than .05 was considered statistically significant. Tabulation and plots of measurements were made with GraphPad Prism^®^ (San Diego, CA, USA).

## Results

Between 2015 and 2019, we found 10 patients submitted to N-MRCP, 7 women and 3 men, with ages between 15 and 61 years, with a median age of 33. Of the 10 patients studied, 5 suffered from recurrent pancreatitis in their medical history, 1 with an asymptomatic elevation of serum amylase and lipase, and 4 patients with recurrent abdominal pain with clinically suspected biliopancreatic origin. At the baseline MRCP, none of them had clear-cut pathological findings and we failed to demonstrate any possible etiology for their medical condition.

**Figure 1.**
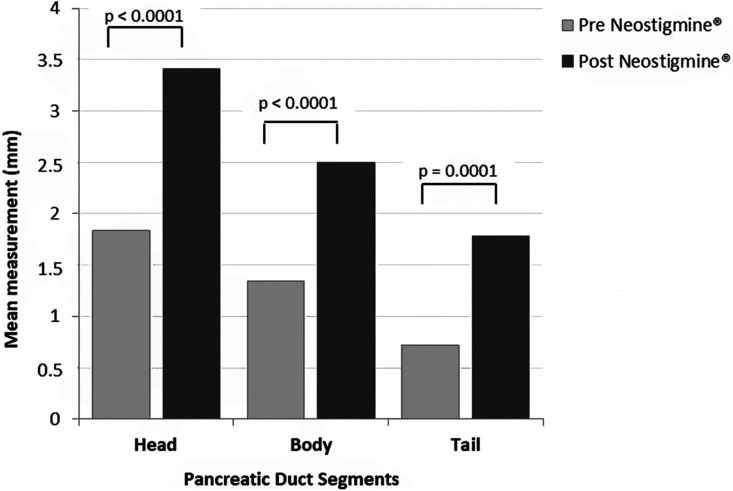
The bar graph shows the mean diameter of the main pancreatic duct in the head, body, and tail before and after 40 minutes of Neostigmine^®^ administration. Grey bars: before Neostigmine^®^, Black bars: after Neostigmine^®^.

The diameter of the PD increased significantly after Neostigmine^®^ in all patients and in all pancreatic segments. Head: 1.84 ± 0.98 mm to 3.41 ± 1.27 mm, increment 85%, *P* < .0001, body: 1.34 ± 0.42 mm to 2.5 ± 0.49 mm, increment 86%, *P* < .0001, and tail: 0.72 ± 0.52 mm to 1.78 ± 0.43 mm, increment 147%, *P* = .0001 (Figure 1).

The basal mean ICC was 0.629 showing good agreement between observers and the post Neostigmine^®^ interobserver ICC was 0.772, showing excellent agreement between the 2 radiologists (Table 2).

**Figure 2.**
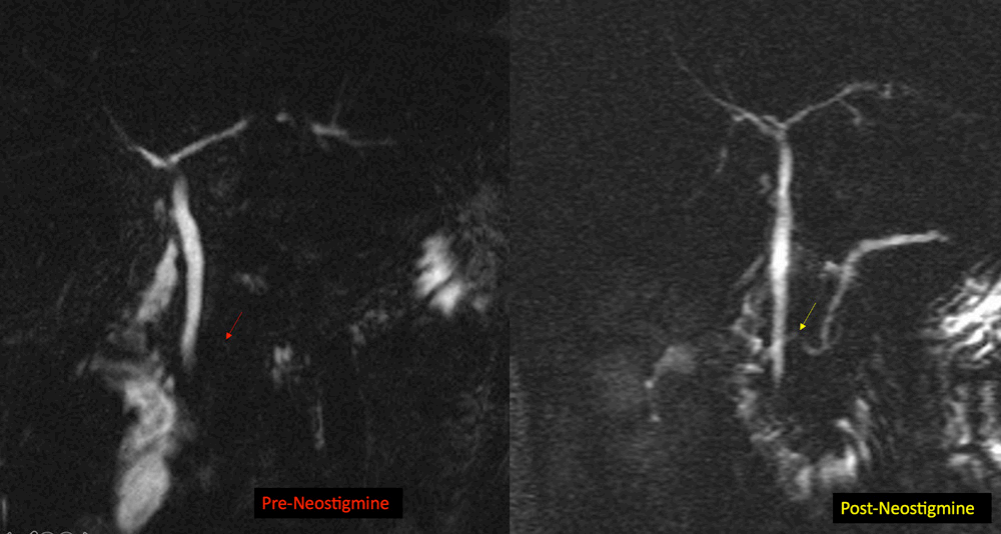
Coronal dynamic magnetic resonance cholangiopancreatograms were obtained before and 40 minutes after Neostigmine^®^ administration in a 33-year-old woman with a history of recurrent acute pancreatitis. In the baseline cholangiographic sequences, the Wirsung duct is not visualized (red arrow). Neostigmine^®^ allows the visualization of the main pancreatic duct (yellow arrow), crossing the choledochus and leading into the minor papilla. The short duct that drains the dorsal anlage of the pancreatic head, continues to be not visualized. These findings confirm pancreas divisum.

**Figure 3.**
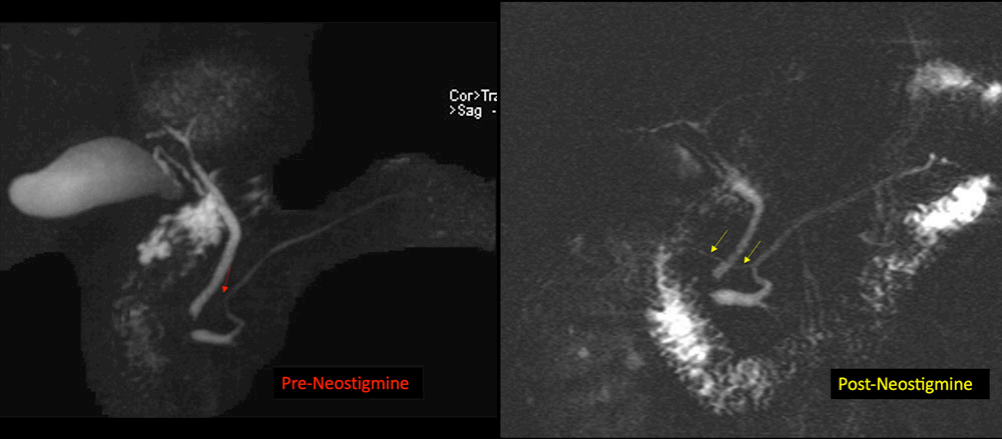
Coronal image from MR cholangiopancreatography of a 61-year-old male patient with a history of recurrent pancreatitis. In baseline cholangiographic sequences, the accessory duct (Santorini) is not seen (red arrow). After administration of Neostigmine^®^, the main pancreatic duct diameter is increased and the Santorini duct is clearly represented (yellow arrows) connected to the main pancreatic duct.

**Figure 4.**
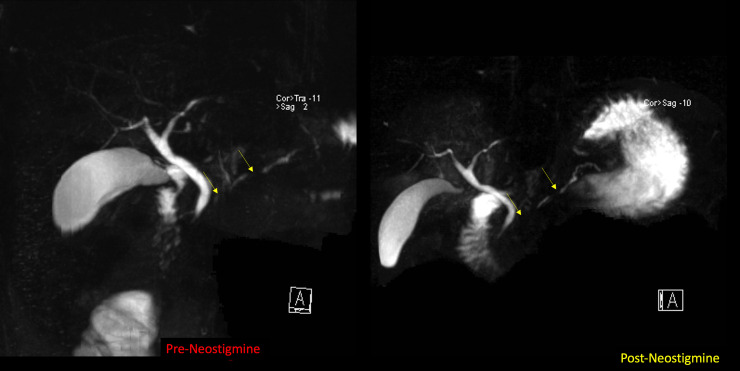
MRCP in a 21-year-old female patient, with a history of recurrent acute pancreatitis. In coronal cholangiographic sequences, there are probable foci of segmental stenosis of the Wirsung (yellow arrows) which do not distend after Neostigmine^®^ administration and become more evident. Note the slight but more pronounced upstream dilatation. The diagnosis of the suspected autoimmune pancreatitis was subsequently confirmed by contrasted images, serology, and response to steroid therapy.

In 2 patients with recurrent pancreatitis, we were able to demonstrate the presence of anatomical variants after administration of Neostigmine^®^, which had not been visualized in the baseline images, confirming pancreas divisum (Figure 2). In another patient, the Santorini duct was not seen on the baseline MRCP images but it was clearly visualized after Neostigmine^®^ administration (Figure 3). In a fourth patient with a history of recurrent acute pancreatitis of uncertain etiology, the administration of Neostigmine^®^ permitted the clear visualization of multiple stenotic segments of the main PD (Figure 4), which were already suspected in the baseline image.

No drug-related side effects were observed in any patient.

## Discussion

The main finding of our study is the confirmation that Neostigmine^®^ injection improved the visualization of pancreatic ducts, and in selected cases, allowed to detect clinically important alterations.

A major diagnostic challenge occurs when patients have a history of acute or recurrent acute pancreatitis with unknown etiology, in the absence of demonstrable pancreatic abnormality on the common image modalities such as MRCP. In our experience, the use of Neostigmine^®^ in MRCP improved the visualization of the full length of the main PD resulting in a statistically significant increase in the diameter of all PD segments. As detailed above, ductal anomaly was confirmed in 2 patients and excluded in a third one. In a fourth patient, N-MRCP improved visualization of multiple stenoses of the main PD, suggesting the possibility of autoimmune pancreatitis, which was later on confirmed based upon imaging findings after Gadolinium administration, elevated IgG4 serum level, and response to corticosteroid therapy.

It is, therefore, that the administration of Neostigmine^®^ changed the treatment strategy in 4 of our 10 patients and also improved the visualization of PD in the other 6 patients.

In contrast with S-MRCP, we did not find this method suitable for the estimation of pancreatic function since cholinergic stimulation increases intestinal motility and quick duodenal emptying interferes with the pancreatic fluid accumulation in the duodenum. Thus, Neostigmine^®^ could replace secretin in 2 aspects: better visualization of the PD system and precise detection of duct anomalies. S-MRCP is performed only in few centers due to its high cost and limited availability. The cost of 1 vial of Secretin is variable between countries, at least 120 USD, and more than 200 USD in others. In contrast, the price of 1 vial of 0.5 mg of Neostigmine^®^ in Chile is less than 1 USD. In addition, the availability of secretin is limited in the majority of countries. In Chile, and probably in several other countries, these factors make unsuitable the use of secretin, even in exceptional cases.

The PD is not always depicted when stenotic or irregular lesions occur without significant dilatation of the pancreatic ducts.^10^ In fact, conventional MRCP can show the main PD in the head, body, and tail of the gland in only 79%, 64%, and 53% of the patients, respectively.^11^ Secretin stimulates the exocrine pancreatic secretion also increases transiently the tone of the sphincter of Oddi,^12^ S-MRCP has emerged as a useful adjunct in assessing pancreatic disorders with improved detection of morphologic abnormalities of the pancreatic duct and evaluating acute and chronic pancreatitis.^6-8^

For this purpose, several authors have proposed the administration of other pharmacological agents able to contract the Oddi sphincter; such action could improve image quality by increasing the pressure and distention of the biliary and pancreatic ducts. Acetylcholine is a potent physiological stimulant of the pancreatic fluid and enzyme secretion and provokes Oddi sphincter contractions.^12^ Neostigmine^®^ is a reversible inhibitor of the enzyme acetylcholinesterase, which hydrolyzes the neurotransmitter ACh, raising ACh concentrations at nerve terminals. Its effect is brief, no more than 4 hours, it is widely used in everyday medical practice, in the postoperative recovery rooms, in Ogilvie syndrome, and in the treatment of myasthenia gravis. Its side effects are rare and mild,^13^ and we did not observe any adverse effects at all in our patients.

The prostigmine–morphine test has been employed in clinical practice as a screening tool in patients with symptoms suggestive of the sphincter of Oddi dysfunction,^14^ while its specificity has been seriously questioned.^15,16^ It was also combined with quantitative hepatobiliary scintigraphy where longer permanence of the radioisotope was demonstrated in the choledochus.^17^ It has been suggested that the administration of morphine–Neostigmine^®^ enhances ductal distension and helps in the differentiation of anatomical variants of the pancreaticobiliary tree on MRCP.^18^ There are contradictory studies regarding the use of opioids alone; Silva et al^19^ described improvement of image quality after administration of morphine by causing sphincter of Oddi contraction. Agarwal et al^20^ demonstrated that fentanyl, a synthetic opioid, improved qualitative and quantitative visualization of the pancreatic and biliary ductal system anatomy. In contrast to these results, Breiman et al^21^ and Kröpil et al^22^ found that opioids had no essential influence on image quality and did not increase bile duct caliber or improve biliary visualization in computed tomographic cholangiography and MRCP, respectively. Chowdhury et al^18^ compared the pancreatic duct morphology following morphine–Neostigmine^®^ and secretin provocation in healthy subjects, where the effects of morphine–Neostigmine^®^ were more pronounced than those of secretin where the mean (SEM) percentage increase in PD diameter was 28.7 (7.2) vs 12.9 (3.3); *P* < .0001, and visible PD length was 49.4 (11.5) vs 28.1 (8.2); *P* < .0001, respectively. In comparison to our results, with the use of Neostigmine^®^ alone, we had an even higher percentage increase in the PD diameter of 85%, 86%, and 147% for the head, body, and tail segments, respectively.

To the best of our knowledge, this is the first study that uses Neostigmine^®^ alone for this purpose. One of the major limitations of the study was the small size of the patient population. MRCP is an excellent method and the visualization of pancreatic ducts is satisfactory in the overwhelming majority. We used Neostigmine^®^ only in cases when the first MRCP did not permit confirmation or exclude a clinically relevant anatomical alteration and we did not include healthy subjects. Another limitation was that we did not examine the effect at different doses of Neostigmine^®^ and different time intervals after the injection and the 2 radiologists who measured the pancreatic duct segments were not blindfolded. However, despite these limitations, we were able to demonstrate the benefits of Neostigmine^®^ administration in improving the visualization of the pancreatic ducts in MRCP images in all cases when it was employed.

In conclusion, the present study demonstrates that administration of Neostigmine® prior to MRCP improves the imaging of pancreatic ducts that may be of value in clinical diagnosis. Its administration increases the diameter of the PD by stimulating pancreatic secretion and thus results in a transient increase of the main PD diameter, which improves its visualization and evaluation in MRCP, making it more precise in its measurement. Neostigmine^®^ is a well-known drug, it is safe, widely available, cheap, and could be used as an alternative to Secretin at an incomparably lower cost. Further prospective studies with a major number of patients can precise the best time interval between Neostigmine^®^ injection and obtaining MRCP images. However, based on our experiences, we recommend its routine use in selected cases in order to improve diagnostic accuracy in the detection of suspected morphological alterations of pancreatic ducts.

## Figures and Tables

**Table 1.** MRCP Imaging Protocol

*MRCP Protocol*
T2 HASTE coronal, 30 slices 3-3.5 mm thick (covering pancreas and bile duct), using IPAT, Grappa and with phase direction in the Head–Feet direction (H–F)
Axial T2 HASTE should cover the entire liver, pancreas, and bile duct, with 40 sections of 3.5- mm thickness, using IPAT, Grappa, and with the phase in the Antero-Posterior direction (A-P)
T2 HASTE sagittal, oriented to the distal common bile duct, with 20-24 slices of 3-3.5 mm thickness, with phase in H-F direction.
Thin axial HASTE T2, spanning from the distal bile duct to the bifurcation of the hepatic ducts and the Wirsung duct, in 30-40 slices of 3 mm thickness, using IPAT, Grappa, and with A-P phase.
T2 HASTE Coronal Oblique, oriented to the distal common bile duct, using the newly acquired axial and sagittal planes. 20-24 sections 3-3.5 mm thick, with H-F phase.
T2 Cholangio3D (volume), with the same orientation as for the Oblique Coronal HASTE. A block of 44 partitions 1.5 mm thick is made.
T2 Fat Sat Axial covering the entire liver and bile duct. 24-30 cuts of 6-7 mm thick are made, with phase in direction A-P and IPAT, Grappa.
T2 Cholangio Thick Slab: 7 radii 50 mm thick are made in relation to the distal common bile duct, requesting the patient to do expiratory apnea.
T2 Dynamic Cholangio: 10 cuts of 30 mm thickness are made in the same position, the one chosen from the radii previously made. The patient is asked to do expiratory apnea.
T1 In - Opp phase Axial, 24-30 slices 5.5-6 mm thick, covering the entire liver and pancreas. The multi breath-hold technique is used, iPAT, GRAPPA, A-P phase direction.

MRCP, magnetic resonance cholangiopancreatography.

**Table 2.** Interclass Correlation Coefficient Between the 2 Radiologists’ Measurements of the Pancreatic Duct Diameter

**Measurements**	**Interclass Correlation Coefficient**	**95% CI**	
**Lower Bound**	**Upper Bound**
Basal			
Head	0.79	0.31	0.95
Body	0.53	0.05	0.85
Tail	0.56	0.004	0.86
Post-Neostigmine®			
Head	0.94	0.79	0.98
Body	0.92	0.72	0.98
Tail	0.45	−0.27	0.83
